# Convulsive Tic

**Published:** 1909-10-02

**Authors:** 


### Diseases of Children.

### CONVULSIVE TIC.

A tic is a co-ordinated purposive act of voluntary type, provoked in the first instance by some external cause or by an idea; repetition leads to it becoming habitual and, finally, to its involuntary reproduction without cause and for no purpose, at the same time as its form, intensity and frequency are exaggerated. It thus assumes the characters of a convulsive movement, inopportune and excessive; its execution is often preceded by an irresistible impulse, resisted at first and then overwhelming and followed by misery at giving way to it, its suppression associated with malaise. The effect of distraction or of volitional effort is to diminish its activity; in sleep it disappears. It occurs in predisposed individuals, who usually show other indications of mental instability. Such is the definition or description, slightly modified, given by Meigs and Feindel in 1908. Tics are often spoken of as "habit spasms," because they are habitual; but it is not a good name, for they are not true habits, and a spasm cannot be controlled by an act of will. Guthrie speaks of them as motor manifestations of psychical unrest or cortical irritability. In a way they are an emotion of expression; expressive gestures preceded by a desire to perform them, and followed by a feeling of satisfaction. The expression varies from a simple twitch of a single muscle to complicated co-ordinated movements.

The "Tic Convulsif," or "Gilles de la Tõurette's Disease," is far the most serious variety. It is a psychical tic with no motor manifestations. Sometimes tic convulsif is regarded as simple tic in a severe form, and the psychical tic as a further stage, which may or may not be associated with motor manifestations. These psychical tics have received various names according to their special characteristics. In the exclamatory tics there are explosive utterances, articulate or inarticulate, which may take the form of echolalia or repetition of words and phrases; coprolalia, or the use of obscene words and bad language; or the cry of some animal. In another form there are fixed ideas, the *folie de doubte*, and various phobias. Some cases are characterised by impulsive acts, and in others there is general mental impairment. These cases generally begin at 10 to 15 years of age, run a progressive course with frequent intermissions, and often end in insanity. Recovery is rare.

An interesting case of this type was reported by Dr. T. W. Edmonston Ross (*British Medical Journal*, 1909, II, 135). The boy was 11 years old, unusually intelligent, and of a somewhat serious disposition. There was no family history of neurosis. Two years previously he exhibited jerky movements in an attack of varicella, and in the following year he had otitis media and very definite tic, with loud hiccoughing sounds or a sharp, squeaking cry during the attacks of spasm. A month later he had scarlet fever, during which the tic was less, but did not entirely cease. He was worse on recovery from the fever, and six months later the explosive utterances, articulate and inarticulate, became prominent, and included hiccoughing sounds and others imitative of dogs, cats and pigs. Exclamations such as dash, bosh, go on, and damn, were shouted, often with wearisome persistency. They usually, but not always, accompanied the muscular spasms. For the next six months the condition varied in severity from week to week, but on the whole got worse, though the boy remained well and even improved in health. The spasms were often forcible, and he would even injure himself with his clenched fist. Walking was interfered with, progression being made by a series of jumps. Loud and incessant shouting became an intolerable trouble. The exclamations included extremely obscene language, and he frequently gave involuntary expression to the thoughts passing in his mind. The moral character deteriorated, and he became untidy, neglectful of his personal appearance, irritable, and disobedient, but had developed no mental obsessions. The most striking feature in this case is the absence of any apparent cause in the family or past history of the child. In all other respects it conforms with the usual type of this rare and interesting affection. The common history is one of severe illness or mental shock in a child of neuropathic heredity. It is difficult to realise that this disease is merely an exaggerated form of the simple "habit spasm" so often seen in the early years of life, notably from five to nine years of age. Even in these mild varieties there are frequently associated signs of nerve irritability or instability, such as timidity, somniloquence, somnambulism, pavor nocturnus, enuresis and emotionalism. In these, however, the prognosis is distinctly good, especially if some exciting cause of the primary spasm can be found and cured. Blepharospasm may be started by errors of refraction or phlyctenules.

The Chorea Electrica of Dubini (1849) affects children of both sexes, at 9 to 15 years of age, and is characterised by sudden muscular contractions of the neck and shoulders. This should hardly be regarded as a special variety. Some of the cases are instances of simple habit spasm; in others there is an associated hysterical element; and in a third type there is a grave lesion of the cortex, and petit mal or Jacksonian epilepsy. It is possible that the patient under consideration also has some cerebral mischief. Encephalitis can occur from infection in chickenpox or other infective fever, and one is inclined to suggest that some cerebral disease of this nature began during the attack of varicella, affected the motor area and the frontal region, and has been followed by slowly progressive cerebral degeneration. It is difficult to believe that these cases are merely exaggerations of the simple variety without any added factor.

The treatment is unsatisfactory. The affection must be carefully diagnosed from hysteria and treated as a nervous disease liable to end in insanity. This involves peace and quiet, no emotional excitement, no mental strain, and a simple country life. Bromides and valerian may be of some use, but little benefit can be hoped for from drugs, if the view of the writer is accepted that there is actual degenerative cerebral mischief.

